# Preservation of Erectile and Ejaculatory Functions After Tetramodal Bladder-Sparing Therapy Incorporating Consolidative Partial Cystectomy Against Muscle Invasive Bladder Cancer

**DOI:** 10.5152/tud.2023.22214

**Published:** 2023-05-01

**Authors:** Yusuke Uchida, Minato Yokoyama, Motohiro Fujiwara, Yuki Nakamura, Yudai Ishikawa, Shohei Fukuda, Yuma Waseda, Hajime Tanaka, Soichiro Yoshida, Takeo Fujiwara, Yasuhisa Fujii

**Affiliations:** 1 Department of Urology, Tokyo Medical and Dental University, Bunkyo-Ku, Tokyo, Japan; 2 Department of Insured Medical Care Management, Tokyo Medical and Dental University, Bunkyo-Ku, Tokyo, Japan; 3 Department of Global Health Promotion, Tokyo Medical and Dental University, Bunkyo-Ku, Tokyo, Japan

**Keywords:** Bladder-sparing therapy, erection, ejaculation, male sexual function, muscle invasive bladder cancer

## Abstract

**Objective:**

To cross-sectionally assess erectile and ejaculatory functions after tetramodal bladder-sparing therapy consisting of transurethral resection, chemoradiotherapy, and consolidative partial cystectomy in patients with muscle invasive bladder cancer.

**Materials and Methods:**

Among 72 enrolled male patients who underwent tetramodal bladder-sparing therapy from 2006 to 2019, 42 who visited the outpatient clinic from February to October 2020 received questionnaires. Erectile function, ejaculatory function, and quality of life were assessed using the International Index of Erectile Function short form, the Male Sexual Health Questionnaire Ejaculatory Dysfunction short form, and the Functional Assessment of Cancer Therapy.

**Results:**

Among the 42 patients, 9 were excluded because of incomplete responses and 33 were eligible for analyses. The median (range) age at survey and the time from treatment completion to responding to the questionnaires was 70 (50-87) years and 4.2 (0.4-14.0) years, respectively. The median International Index of Erectile Function short form-5 score was 11 (5-25), and 3 (9.1%) and 9 (27.3%) patients had no and mild erectile dysfunction, respectively. The Male Sexual Health Questionnaire Ejaculatory Dysfunction short form results showed that 23 (69.7%) patients responded that they could ejaculate. Patients with higher Male Sexual Health Questionnaire Ejaculatory Dysfunction short form scores had better erectile function and quality of life than those with lower Male Sexual Health Questionnaire Ejaculatory Dysfunction short form scores.

**Conclusion:**

Preservation of erectile and ejaculatory functions was demonstrated in muscle invasive bladder cancer patients treated with tetramodal bladder-sparing therapy. In addition to lower urinary tract function, preservation of male sexual function, especially ejaculatory function, in bladder-sparing therapy can be an advantage over radical cystectomy.

**Main Points**

- Radical cystectomy, the gold standard treatment for muscle invasive bladder cancer (MIBC), inevitably impairs male sexual function.
- Sexual function after tetramodal bladder-sparing therapy (TeMT) which consists of bladder tumor transurethral resection, chemoradiotherapy, and consolidative partial cystectomy has not been evaluated.
- Patient-reported outcomes indicated that erectile and ejaculatory functions were preserved in most MIBC patients treated with TeMT.

## Introduction

Radical cystectomy (RC) is the gold standard treatment for nonmetastatic muscle invasive bladder cancer (MIBC). In addition to the bladder, the prostate is simultaneously removed when male patients undergo RC. Therefore, RC results in loss or impairment of lower urinary tract function and has a significant negative impact on male sexual function.^1-3^ Even nerve-sparing RC, which can preserve erectile function, necessarily inhibits ejaculatory function. Bladder-sparing therapy (BST) was reported to achieve good cancer control and quality of life (QOL) after treatment, and BST for MIBC is recognized as an optimal treatment for selected patients.^4,5^ However, studies on sexual dysfunction after bladder cancer treatment have been biased toward the post-RC population, and there are few studies on male sexual function after BST.^6^

The current main BST protocol is trimodality therapy (TMT), consisting of transurethral resection of bladder tumor (TURB) and chemoradiotherapy (CRT). However, MIBC recurrence after BST remains a clinical challenge. Previous studies have shown that even if complete MIBC remission was achieved using TMT, the MIBC recurrence rate is 11-19%,^7-9^ and the site of recurrence is often the original MIBC site. Nodal recurrence after BST is also a clinical problem.^9,10^ To reduce the risk of local and nodal recurrence, Kihara et al^11-13^ developed tetramodal bladder-sparing therapy (TeMT) incorporating consolidative partial cystectomy (PC) with pelvic lymph node dissection in addition to maximal TURB and induction CRT. Although they previously reported that patients undergoing TeMT have good lower urinary tract function,^13^ sexual function after TeMT has not been evaluated. Tetramodal bladder-sparing therapy including consolidative PC that does not damage the prostate can spare erectile and ejaculatory functions. The purpose of this study was to cross-sectionally assess erectile and ejaculatory functions and QOL after TeMT in male MIBC patients using patient-reported outcomes (PRO).

## Materials and Methods

### Patients

The Ethics Committee of Tokyo Medical and Dental University approved the current study (approval number: M2000-453-01). Among 117 male Japanese patients with nonmetastatic MIBC (T2-3N0M0) who completed TeMT at our institution between July 2006 and November 2019, 45 patients who underwent RC for intravesical recurrence or systemic chemotherapy for distant metastasis or MIBC recurrence, had a history of treatment for prostate cancer, or were censored due to loss to follow-up or death were excluded. Among the remaining 72 enrolled patients, 42 visited the outpatient clinic from February to October 2020 and received questionnaires. All the patients provided written informed consent.

### Treatment Protocol

**Figure 1.**
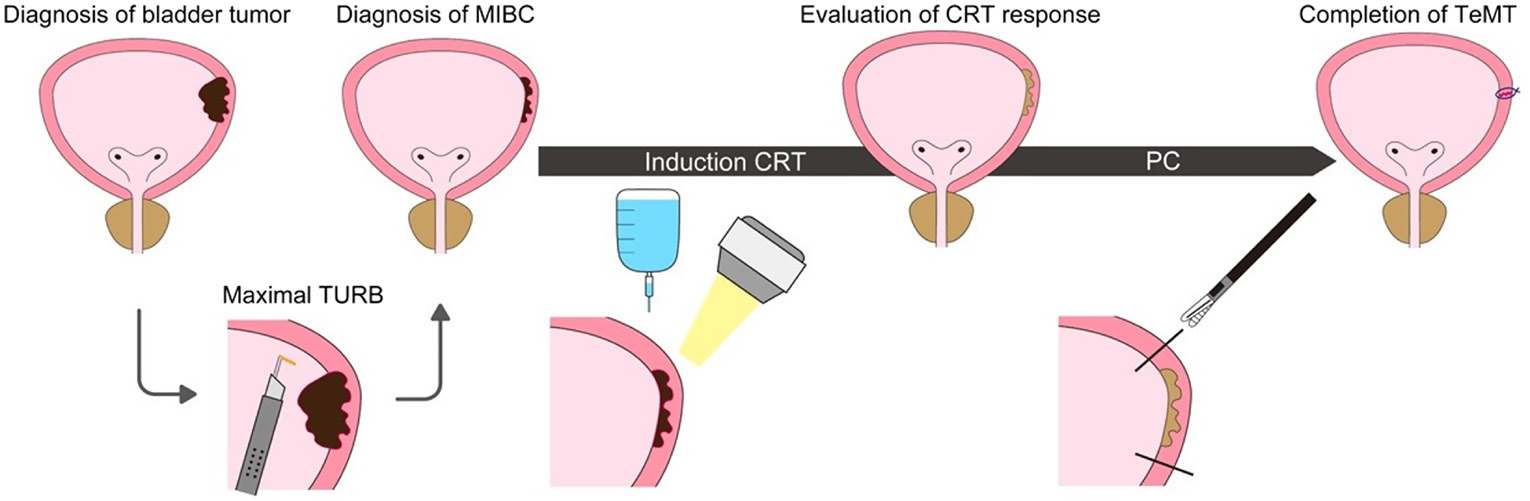
Schema of TeMT. CRT, chemoradiation therapy; MIBC, muscle invasive bladder cancer; PC, partial cystectomy; TeMT, tetramodal bladder-sparing therapy; TURB, transurethral resection of bladder tumor.

The tumor characteristic-based inclusion criteria for the TeMT were as follows: pathologically confirmed urothelial carcinoma; tumor occupying less than 25% of the bladder surface; absence of bladder neck involvement; and absence of broad carcinoma in situ. The TeMT protocol starts with maximal TURB and induction CRT (external beam radiotherapy to the true pelvis with 40 Gy in 20 fractions and 2 cycles of concurrent intravenous cisplatin 20 mg for 5 days separated by 3-week intervals), which is followed by consolidative PC with pelvic lymph node dissection after complete MIBC remission is confirmed (Figure 1).^12,13^

### Questionnaires

Erectile function, ejaculatory function, and QOL were assessed using the International Index of Erectile Function short form (IIEF-5), Male Sexual Health Questionnaire Ejaculatory Dysfunction short form (MSHQ-EjD-SF), and the Functional Assessment of Cancer Therapy-Bladder (FACT-Bl), respectively.^14-16^ The questionnaires used in this study were all Japanese versions. The IIEF-5 is a 5-item questionnaire, and its scoring procedure is the sum of the responses of all 5 items. The possible IIEF-5 scores range from 5 to 25. The MSHQ-EjD-SF is a 4-item questionnaire. The scoring procedure is divided into an ejaculatory function score, which is the sum of the responses to the first 3 items (frequency, volume, and force of ejaculation) and the bother/satisfaction score, which is the response to the independent fourth item. The ejaculation assessment in this study was conducted by extracting the ejaculatory function score from the MSHQ-EjD-SF. The possible ejaculatory function scores range from 1 to 15, and a higher score indicates better ejaculatory function. The FACT-Bl is a 39-item questionnaire to measure QOL of bladder cancer patients, which consists of the FACT-G questionnaire (a generic QOL measure) and 12 bladder-specific items. It is divided into the following 5 domains: physical well-being (PWB); social/family well-being (SWB); emotional well-being (EWB); functional well-being (FWB); and additional concerns as a bladder cancer subscale (BlCS). All items are scored on a 5-point scale (0-4), and a higher score indicates a better QOL on all subscales and items. In the FACT-Bl, sexual questions were asked on GS7 (“I am satisfied with my sex life”), BL4 (“I am interested in sex”), and BL5 (“I am able to have and maintain an erection”). Scoring and handling of missing data were based on the FACT-Bl scoring guideline and the Functional Assessment of Chronic Illness Therapy measure.^17^

Attending physicians gave the questionnaires to the patients during the first outpatient visit between February 2020 and October 2020. The patients completed them outside the office and returned them to the medical assistants without a physician present. Japanese translation of the MSHQ-EjD-SF did not undergo the full linguistic validation process, while the validated IIEF-5 and FACT-Bl were translated into Japanese using an iterative forward-backward translation method.^18,19^

### Statistical Analysis

Descriptive statistics were performed using Fisher’s exact test for categorical variables and the Mann–Whitney *U*-test for continuous variables. All *P* values were 2-tailed, and *P* < .05 was considered statistically significant. All statistical analyses were performed using JMP v10.0.2 (SAS Institute, Cary, NC, USA).

## Results

**Figure 2.**
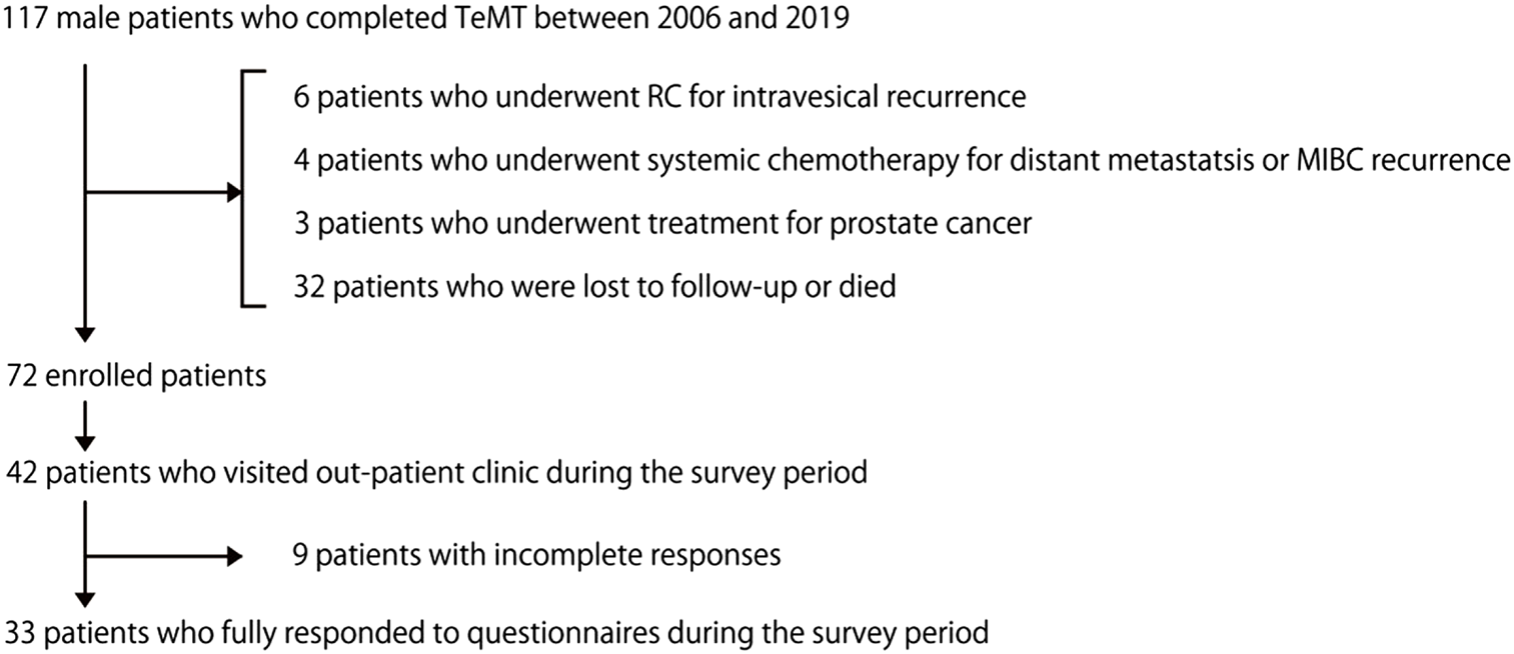
Study flowchart. MIBC, muscle invasive bladder cancer; RC, radical cystectomy; TeMT, tetramodal bladder-sparing therapy.

Among the 42 patients who received a questionnaire, 33 (79%) patients fully completed it (Figure 2). The characteristics of the 33 patients were shown in Table 1. The median (range) age of the 33 patients was 70 (50-87) years, and the time from TeMT completion to the date of response was 4.2 (0.35-14.0) years. Comorbidities that might affect male sexual function were as listed in Table 1, and 11 of the 33 patients had no comorbidities.

The median IIEF-5 score was 11, and 3 (9.1%), 9 (27.3%), 4 (12.1%), 4 (12.1%), and 13 (39.4%) had no erectile dysfunction (ED) (22-25 points), mild ED (17-21 points), mild to moderate ED (12-16 points), moderate ED (8-11 points), and severe ED (5-7 points), respectively (Table 2). Based on the results of MSHQ-EjD-SF item 1, ejaculatory function was preserved in 23 patients (69.7%), although the other 10 patients could not ejaculate (Table 3). The median ejaculatory function score was 6. The median score for item 4 was 1, which indicated that most of the patients were not bothered by their own ejaculatory function. Scores on the FACT-Bl are shown in Table 4. The median FACT-G values and the total score were 80 and 117.2, which represented 74% and 75% of the scale range, respectively. The median (range) of GS7, BL4, and BL5 were 2 (0-4), 2 (0-4), and 2 (0-4), respectively.

**Figure 3.**
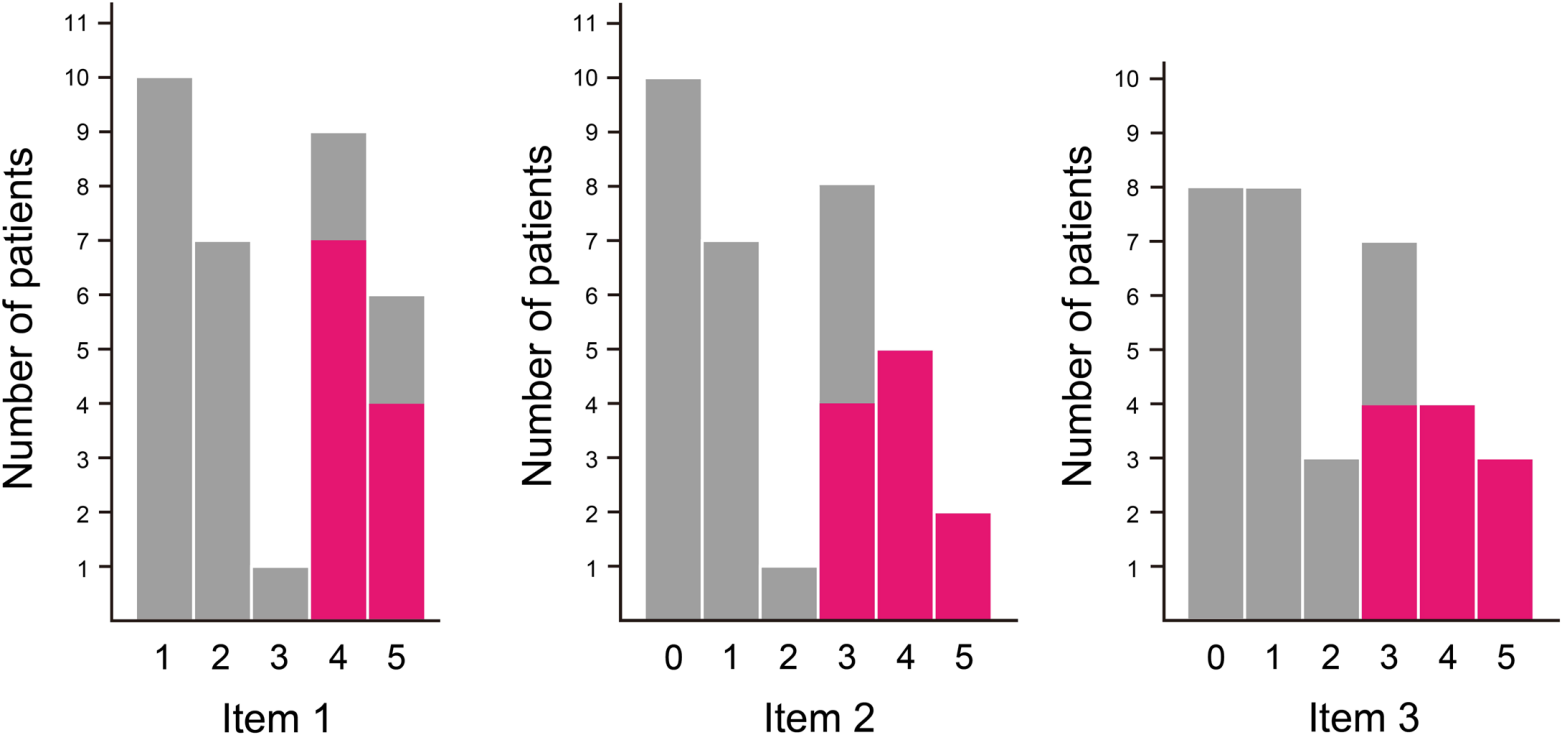
Distribution of MSHQ-EjD-SF item scores. The colored area indicates the number of patients classified as MSHQ-EjD-SF high scorers. MSHQ-EjD-SF; Male Sexual Health Questionnaire Ejaculatory Dysfunction short form.

The MSHQ-EjD-SF item 1-3 score distributions were bimodal, as shown in Figure 3. We then defined MSHQ-EjD-SF high scorers as patients who responded with a score of 4 or greater on the MSHQ-EjD-SF item 1, and 3 or greater on items 2 and 3. Eleven of 33 (33%) patients were classified as MSHQ-EjD-SF high scorers, and the other 22 (67%) were classified as MSHQ-EjD-SF low scorers. The MSHQ-EjD-SF high scorers were significantly younger and had higher renal function than MSHQ-EjD-SF low scorers (Table 5). They also had less cardiovascular disease and hypertension. The IIEF-5 total score for the MSHQ-EjD-SF high scorer was significantly higher than that of the MSHQ-EjD-SF low scorer. In the FACT-Bl assessment, MSHQ-EjD-SF high scorers recorded a significantly higher score than MSHQ-EjD-SF low scorers on all subscales except for PWB and SWB. Similarly, GS7, BL4, and BL5 as sexual question items were significantly higher in the MSHQ-EjD-SF high scorer than in the MSHQ-EjD-SF low scorer (median 3 vs. 1, 3 vs. 2, and 3 vs. 0, respectively, all *P* < .01).

## Discussion

In the present study, we cross-sectionally evaluated erectile and ejaculatory functions after TeMT on the basis of PROs. Our results showed that more than one-third of Japanese male patients treated with TeMT, which was a type of BST incorporating consolidative PC, had no or mild ED and more than two-thirds could ejaculate. Compared with the FACT-G score in a German-speaking general population,^20^ our patients undergoing TeMT had comparable scores for PWB, EWB, and FWB. The generic QOL score for the MSHQ-EjD-SF high scorer and the results of the FACT-Bl were significantly better than those of the MSHQ-EjD-SF low scorer, suggesting that preservation of sexual function might affect the QOL.

Radical cystectomy, which is still the gold standard for localized MIBC treatment, greatly impairs male sexual function.^21^ A previous study reported that only 8% of the male patients achieved a sufficient erection for intercourse after RC, and none of them were able to ejaculate.^22^ Although the degree of erectile function impairment is affected by the preservation of neurovascular bundles,^23^ the loss of ejaculatory function is practically inevitable. Exceptional preservation of ejaculatory function can be achieved by a seminal tract-sparing cystectomy, which is not commonly performed.^24^ A large prospective cohort study showed that RC performed at a high-volume center did not lead to permanent decreases in all QOL domains except for sexual function.^25^ Sex was reported to be what made people feel happiest,^26^ and its loss would have a significant negative impact on the QOL.

Unlike RC, TMT may preserve sexual function because the seminal tract is spared. However, there are limited studies on the evaluation of sexual function after TMT. A previous cross-sectional study based on a generic QOL questionnaire reported that patients with TMT showed better erectile and ejaculatory functions and were less uncomfortable with being sexually intimate than those with RC, and there was a large difference in the scores.^27^ Another cross-sectional study regarding male sexual function after TMT demonstrated that 54% of the patients reported a sufficient erection for vaginal penetration, and 50% were capable of ejaculation.^28^ Preservation of both lower urinary tract function and sexual function would contribute to a good QOL for patients undergoing TMT.

Because consolidative PC in the TeMT protocol does not damage the prostate or urethra, sexual function is expected to be preserved as in TMT. In the current study, we reported that 36% of patients had normal erectile function or mild ED and that 70% were able to ejaculate. To the best of our knowledge, this is the first report of a PRO assessment of the quality of erection and ejaculation after BST. Our IIEF-5 results were comparable with those of a previous report by Rhoden et al^29^ in which approximately 40% of healthy men in their 70s had no or mild ED. Chemoradiotherapy with or without PC may be less likely to cause clinically significant damage to erectile function. Rosen et al^15^ developed the MSHQ-EjD-SF questionnaire and reported that the median ejaculatory function score (sum of items 1-3) in over 1000 men with lower urinary tract symptoms was 13. Their median age was comparable to that of our cohort, but the median ejaculatory function score in TeMT patients was as low as 6. Because reference scores for MSHQ-EjD-SF in Japanese populations have not been reported and the pretreatment ejaculatory status for our cohort was not available, it is difficult to assume the impact of TeMT on ejaculatory function. The MSHQ-EjD-SF high scorers who were characterized as a young and healthy population comprised one-third of our cohort, and the median ejaculatory function score of 12 was comparable to that of the reference population,^15^ which indicated that they could enjoy sexual activities even after multidisciplinary treatment for MIBC.

There were several limitations in this study. First, because this was a cross-sectional study, baseline data were not available and changes over time could not be analyzed. The time between TeMT completion and the questionnaire survey also varied widely. The second limitation was the small cohort consisting of respondents treated with TeMT, which was not widespread. Preservation of sexual function after TMT with full-dose CRT as the most commonly performed BST remains unclear. However, possible preservation of erectile and ejaculatory function even after TeMT, which is a type of BST incorporating consolidative PC in addition to maximal TURB and induction CRT, is considered to be of great value. Third, this assessment of sexual function was only based on PRO. Objective evaluations are needed to confirm the preserved sexual functions after BST. Future studies are required to determine if CRT or PC has an impact on male sexual function. Another limitation was that we used a non-validated Japanese version of MSHQ-EjD-SF. In daily practice, however, we evaluated the ejaculation functional change before and months after initiation of androgen deprivation therapy for several prostate cancer patients using MSHQ-EjD-SF. The ejaculatory function score was significantly reduced by treatment (data not shown), which indicated that the Japanese version of the MSHQ-EjD-SF was feasible for assessing ejaculatory function.

In conclusion, a considerable number of MIBC patients treated with TeMT, which is a type of BST incorporating consolidative PC, preserved their erectile and ejaculatory functions. In addition to preserving lower urinary tract function, BST is considered superior to RC in preserving male sexual function, which is inevitably impaired by RC.

## Figures and Tables

**Table 1.** Patient Characteristics

Variables	
Age (years)	70 (50-87)
Body mass index (kg/m^2^)	26.3 (19.1-31.2)
Time from PC to response (years)	4.2 (0.35-14.0)
Smoking (current/former/never)	0 (0)/24 (72.7)/9 (27.3)
Initial clinical T stage (2/3)	19 (57.6)/14 (42.4)
Histology (pure UC/other variants)	33 (100)/0 (0)
Comorbidities	
Cerebral infarction	1 (3.0%)
Cardiovascular disease	8 (24.2%)
Neurological disorder	3 (9.1%)
Hypertension	15 (45.5%)
Diabetes mellitus	5 (15.2%)
Depression	2 (6.1%)
Sleep apnea syndrome	1 (3.0%)
Benign prostate hyperplasia	2 (6.1%)

Continuous variables are presented as the median (range), and categorical variables are presented as the number (%).

PC, partial cystectomy; UC, urothelial carcinoma.

**Table 2.** IIEF-5 Item Scores

Item 1		How do you rate your confidence that you could get and keep an erection?	
1 Very low	9 (27.3%)
2 Low	9 (27.3%)
3 Moderate	11 (33.3%)
4 High	1 (3.0%)
5 Very high	3 (9.1%)
Item 2		When you had erections with sexual stimulation, how often were your erections hard enough for penetration?	
1 Almost never/never	11 (33.3%)
2 A few times (much less than half the time)	8 (24.2%)
3 Sometimes (about half the time)	5 (15.2%)
4 Most times (much more than half the time)	7 (21.2%)
5 Almost always/always	2 (6.1%)
Item 3	
During sexual intercourse, how often were you able to maintain your erection after you had penetrated (entered) your partner?	
1 Almost never/never	14 (42.4%)
2 A few times (much less than half the time)	4 (12.1%)
3 Sometimes (about half the time)	5 (15.2%)
4 Most times (much more than half the time)	8 (24.2%)
5 Almost always/always	2 (6.1%)
Item 4	
During sexual intercourse, how difficult was it to maintain your erection to completion of intercourse?	
1 Extremely difficult	14 (42.4%)
2 Very difficult	2 (6.1%)
3 Difficult	2 (6.1%)
4 Slightly difficult	5 (15.1%)
5 Not difficult	10 (30.3%)
Item 5	
When you attempted sexual intercourse, how often was it satisfactory for you?	
1 Almost never/never	14 (42.4%)
2 A few times (much less than half the time)	5 (15.2%)
3 Sometimes (about half the time)	4 (12.1%)
4 Most times (much more than half the time)	6 (18.2%)
5 Almost always/always	4 (12.1%)

IIEF-5, International Index of Erectile Function short form.

**Table 3.** MSHQ-EjD-SF Item Scores

Item 1		How often have you been able to ejaculate when having sexual activity?	
5 All the time	6 (18.2%)
4 Most of the time	9 (27.3%)
3 About half the time	1 (3.0%)
2 Less than half the time	7 (21.2%)
1 None of the time/could not ejaculate	10 (30.3%)
Item 2		How would you rate the strength or force of your ejaculation?	
5 As strong as it always was	2 (6.1%)
4 A little less strong than it used to be	5 (15.2%)
3 Somewhat less strong than it used to be	8 (24.2%)
2 Much less strong than it used to be	1 (3.0%)
1 Very much less strong than it used to be	7 (21.2%)
0 Could not ejaculate	10 (30.3%)
Item 3		How would you rate the amount or volume of semen when you ejaculate?	
5 As much as it always be	3 (9.1%)
4 A little less than it used to be	4 (12.2%)
3 Somewhat less than it used to be	7 (21.2%)
2 Much less than it used to be	3 (9.1%)
1 Very much less than it used to be	8 (24.2%)
0 Could not ejaculate	8 (24.2%)
Item 4		If you have had any ejaculation difficulties or have been unable to ejaculate, have you been bothered by this?	
0 No problem with ejaculation	6 (18.2%)
1 Not at all bothered	14 (42.4%)
2 A little bothered	6 (18.2%)
3 Moderately bothered	6 (18.2%)
4 Very bothered	1 (3.0%)
5 Extremely bothered	0 (0%)

MSHQ-EjD-SF, Male Sexual Health Questionnaire Ejaculatory Dysfunction short form.

**Table 4.** FACT-Bl Subscale Scores

	Scale	No. of Items	Score Range	Median Score (Range)
FACT-G	PWB	7	0-28	25 (13-28)
	SWB	7	0-28	13 (2-28)
	EWB	6	0-24	21 (7-24)
	FWB	7	0-28	22 (8-28)
BlCS		12	0-48	34.8 (20.4-48)
Total		39	0-156	117.2(73-148)

BlCS, bladder cancer subscale; EWB, emotional well-being; FACT-Bl, Functional Assessment of Cancer Therapy-Bladder; FACT-G, a generic QOL questionnaire; FWB, functional well-being; PWB, physical well-being; SWB, social/family well-being; QOL, quality of life.

**Table 5.** Comparison of Patient Characteristics Between the MSHQ-EjD-SF High and Low Scorers

Variables	MSHQ-EjD-SF High Scorer (n = 11)	MSHQ-EjD-SF Low Scorer (n = 22)	*P*
Age (years)	66 (52-76)	74 (50-87)	.0241
Body mass index (kg/m^2^)	25.0 (19.9-31.2)	24.3 (19.1-29.4)	.9391
Time from PC to response (years)	6.3 (1.0-14.0)	4.0 (0.35-12.4)	.1364
Smoking (current/former/never)	0 (0)/8 (72.7)/3 (27.3)	0 (0)/16 (72.7)/6 (27.3)	1
Comorbidities			
Cerebral infarction	0 (0)	1 (4.5)	1
Cardiovascular disease	0 (0)	8 (36.4)	.0313
Neurological disorder	0 (0)	3 (13.6)	.5343
Hypertension	2 (18.2)	13 (59.1)	.0342
Diabetes mellitus	2 (18.2)	3 (13.6)	1
Depression	0 (0)	2 (9.1)	.5417
Sleep apnea syndrome	1 (9.1)	0 (0)	.3333
Benign prostate hyperplasia	0 (0)	2 (9.1)	.5417
IIEF-5 total score	19 (13-25)	6 (5-20)	.0001
ED categories			.0006
No ED	3 (27.2)	0 (0)	
Mild ED	5 (45.5)	4 (18.2)	
Mild to moderate ED	3 (27.2)	1 (4.5)	
Moderate ED	0 (0)	4 (18.2)	
Severe ED	0 (0)	13 (59.1)	
FACT-Bl subscales and items			
PWB	25 (21-28)	24.5 (13-28)	.8560
SWB	17 (2-28)	11.3 (4-25)	.1003
EWB	22 (19-24)	20 (7-24)	.0451
FWB	25 (18-28)	20.5 (8-28)	.0170
BlCS	39.6 (28-48)	33.6 (20.4-42)	.0109
FACT-G	87 (71-101)	76.4 (49-96)	.0074
Total	127.8 (104.6-148)	109.2 (73-134.4)	.0091

Continuous and ordinal variables are presented as median (range) and categorical variables are presented as number (%).

BlCS, bladder cancer subscale; ED, erectile dysfunction; EWB, emotional well-being; FACT-Bl, Functional Assessment of Cancer Therapy-Bladder; FACT-G, a generic QOL questionnaire; FWB, functional well-being; IIEF-5, International Index of Erectile Function short form; MSHQ-EjD-SF, Male Sexual Health Questionnaire Ejaculatory Dysfunction short form; PC, partial cystectomy; PWB, physical well-being; SWB, social/family well-being; QOL, quality of life.
